# Medical Opinion and Movement

**Published:** 1910-04-09

**Authors:** 


### Medical Opinion and Movement.

### Acid Dyspepsia and Chlorides.

Dr. Romkes proposes to treat acid dyspepsia by means of a diet free from chlorides. He points out that the usual administration of alkalies is unsatisfactory, as it must eventually give rise to increased formation of acid in the system in order to neutralise the alkalies, so that the dose of alkalies in order to be efficacious should be continually increased. Dr. Romkes finds that the injection of 4 to 6 grammes of sodium chloride in capsules, so that the salt is only absorbed in the intestine, results in a considerable increase of gastric acid secreted, showing that the acid secretion depends in a large measure upon the chloride absorbed with the food. Inversely with five patients suffering from hyperchloridia, feeding with a diet almost free from chlorides was followed almost immediately by a considerable reduction in the acid secretion. He has not observed any ill-effects from deprivation of chlorides in the food, which is of course never absolute. He suggests that the success of certain diets, notably that of Lenhartz, in these gastric conditions is actually due to their deficiency in chlorides. The diet should consist of eggs, starchy foods, and green vegetables, and should be sparse in meat. He gives preference to mutton and veal as containing only a small proportion of chlorides.

### Furuncles treated by Bier's Method.

Bier's method of hyperæmic congestion for all manner of local inflammatory conditions has been so much reported upon and discussed of late that its possibilities, as well as its limitations, are now well recognised by the profession. The treatment of furuncles of the face by this method would appear, however, to offer special difficulties, and a report on a number of cases so treated by Dr. W. Keppler, assistant of Dr. Bier, is not without interest. His report deals with 12 severe cases of furuncles of the upper lip, and 24 cases of a more mild nature, in which the lower lip and other parts of the face were affected. All the cases were cured by the treatment in the course of four to six days. The technique of the treatment is as follows: an elastic band, three centimetres wide, is applied around the neck as low as possible and fixed at the back by a hook and eye. It need only be drawn moderately tight, as stasis is easily produced in the neck with only a moderate amount of constriction. A compress may be placed within the band. The band should be kept on for 20 to 24 hours. The inconvenience experienced soon passes off. The face becomes swollen, and especially the affected parts. At the end of one to three days of hyperæmia the inflamed area softens and suppurates freely, then the discharge diminishes, and is followed by the process of healing. Applications should be made each day, the duration being gradually reduced, till the inflammatory process is at an end and repair of the tissues commences.

### Cerebral Concussion.

An interesting contribution is made by Professor Trendelenburg to the *Deutsche Medizinische Wochenschrift* on cerebral concussion. He draws a comparison between the symptoms of this condition and those of ether or chloroform anæsthesia or acute alcoholic narcosis, and he is led by the analogy to designate it a "traumatic narcosis." In both there is loss of consciousness with more or less diminution of the pupillary reflexes and in both the condition is followed by vomiting. He does not find the pulse is always slowed. On the contrary it is frequently accelerated to 80 or 90, or even 120 a minute. The temperature may be normal or slightly reduced if taken an hour after the accident, but later on it tends to be raised slightly and may remain so for the following day. One of the most interesting features is the retrograde amnesia—the inability of the patient to remember what has happened during or shortly before the accident. The extent and duration of this have a certain relation to the duration of the period of unconsciousness; the shorter the latter the quicker the memory is regained. A loss of consciousness of five to fifteen minutes is sufficient to produce the condition of retrograde amnesia. Sometimes this hiatus in the memory is bridged over by false mental impressions, and this fact may prove of importance from a medico-legal aspect. Thus a person sustaining a fall may assert that he has been run over or has received a blow on the head. In severe cases moreover the patient's capacity for receiving mental impressions may be so reduced for some days after recovery of consciousness that he may be unable afterwards to recall what has happened during this period. The author is inclined to the view that the presence of traumatic psychoses points to a contusion of the brain accompanying the concussion rather than to the concussion alone.

### The Causation of Sex.

In an extremely thorough and valuable paper contributed to the *British Journal of Obstetrics and Gynæcology*, Dr. A. E. Giles considers in detail the after-results of 1,000 consecutive operations upon the pelvic organs. The original paper is well worth consulting on account of the many interesting points considered and investigated. Those who are interested in theories of the determination of sex, and have read Dr. Rumley Dawson's book, which we lately reviewed, in which he advocates the idea that the right ovary produces males only, and the left females, will note especially what Dr. Giles has to say on the subject of pregnancy after removal of one ovary. Twenty-three patients who had undergone this operation subsequently became pregnant and went to term, some of them more than once; 29 children were born to them. Ten women still possessing the right ovary bore between them seven boys, seven girls, and one child whose sex is not recorded.

Thirteen women with left ovaries only were delivered of eight boys, five girls, and one child whose sex is not recorded. In two of these cases the patients, who had but one ovary each (the right in both cases) bore children of different sexes. Another curious fact in relation to pregnancy following removal of a tube and ovary is this: About 25 per cent. of women of child-bearing age thus treated subsequently became pregnant, and among these pregnancies nearly a quarter are extra-uterine. Probably old tubal inflammation on the side not interfered with, co-existent with that for which salpingectomy has been performed, is the explanation of this high proportion.

### Chauffeur's Fracture.

The back kick with which the crank handle of a motor-car may strike the arm or jerk the wrist of the person endeavouring to start the engine has long been known as a common cause of fracture. As a rule the break is due to indirect violence, and it is usually near the lower end of the radius; hence it is generally classified as a Colles' fracture. Dr. Thomas, of New York, who has seen and studied 30 cases of this injury, regards it as a distinct clinical entity differing in several respects from the typical Colles. In the latter, fracture of the styloid process of the ulna is now known, thanks to $x$-rays, to be the rule; 71 per cent. are thus complicated, Dr. Thomas says, and other authorities have published even higher proportions. But in chauffeur's fracture the styloid process is fractured only in about 23 per cent. The prevailing type occurs lower on the radius, with milder symptoms and less displacement than in the usual Colles' injury sustained by falling on the outstretched palm. The fracture is either transverse or oblique into the wrist joint, and the author believes it to be caused by avulsion, not by impact through the palm. The fracture produced by direct violence, when the handle flies round and hits the forearm, is much less common; the radius or ulna, or both, may be broken. All these injuries can be avoided by care not to use the crank handle with the spark advanced, and by grasping it in the fingers and palm only, not using the thumb. Fortunately, treatment on the usual lines is easy and satisfactory in its results.

### Radium Therapy.

In the *Bristol Medico-Chirurgical Journal* Mr. James Mackenzie Davidson deals with some of the therapeutic and other properties of radium, which substance he was the first to employ in Great Britain as a medical measure. That rodent ulcer is well adapted for radium treatment is now fairly widely known, for many radiologists have testified the same fact. It is interesting to note that it was for a case of this disease that the author first tried radium, and that the cure effected more than six years ago has been perfect ever since. On the other hand, carcinoma does not seem to respond to the treatment, even when it has invaded the skin, as, for instance, in spots and nodules of recurrence after malignant disease of the breast. Tuberculosis verrucosa has been cured by the author, and amongst his other successes have been small epitheliomata of the skin and tongue, moles, nævi, and port-wine stains. Perhaps his most interesting observation, however, is his most recent one: for Mr. Davidson finds that radium will cure $x$-ray dermatitis. So far he has only been able to experiment upon small patches of this terrible condition, owing to the small quantity of radium at his command; but if this announcement turns out to be as well founded as he believes, it is evident that no better work awaits the Radium Institute than the healing of those who have incurred this terrible complaint during their researches for the advancement of radiology. At present the author is devoting much time to the investigation of diseases of the eyes and lids; in corneal ulcer, hypopym, episcleritis, pterygium, and granular lids it has already yielded results far superior to those of any other method.

### Calabar Swellings.

The ætiology of this peculiar condition has long been a matter of speculation amongst those who see patients from the West Coast of Africa. In an individual, probably otherwise healthy, an œdematous, non-inflammatory swelling many inches in diameter suddenly and without obvious cause appears on a limb or elsewhere on the body. Without causing much pain it subsides in a few days, and is never known to suppurate. Sir Patrick Manson has lately had the opportunity of puncturing one of these swellings within a few hours of its onset. He has long entertained the hypothesis that the condition is one associated with the presence of *Microfilaria loa*, and he has favoured most the supposition that it is caused by the periodical and normal emptying of the uterus of the gravid female worm into the connective tissues of the host. In the case now published in the *Journal of Tropical Medicine and Hygiene* he punctured such a swelling and obtained two drops of clear serum which he stained with carbol-fuchsin and hæmatoxylin respectively. Unfortunately the patient's peripheral blood swarmed with embryoes, so the possibility that the needle punctured a capillary *en route* cannot be excluded. Still the red blood corpuscles seen in the films were so scanty that it is probable that this did not occur. At any rate, one film showed thirteen, the other seven *Microfilariæ*. He appeals to medical men who see much of this condition to settle the point by examining a patient in whose blood the embryoes cannot be found.

### The Medical Treatment of Whooping Cough.

A plan for the local and internal treatment of whooping cough, originally recommended in Ander's "System of Medicine," has been very favourably reported on by Dr. McEvitt. The usual hygienic rules are observed very carefully, and the drug treatment is as follows: The throat and nose are sprayed regularly with a mixture of equal parts of peroxide of hydrogen and glycerine. If there is any nasal discharge an ointment is applied to the nares every two hours, containing menthol, boric acid, and white vaseline. The tasteless tannate of quinine is given also in small doses; in solution to infants, and in chocolate tablets to children of four years and upwards. These are all ordered for their antiseptic effects. In addition, emulsion of asafœtida (U.S.P.) is given as an anti-spasmodic. This stimulating and anti-spasmodic expectorant is also carminative in action, so much so that it must be administered very carefully or else it will upset the stomach. For an infant under five months the author prescribes four or five drops every two hours, and increases gradually to eight or ten drops. At four years he begins with fifteen and increases to thirty drops. Notwithstanding the objectionable odour of the drug, Dr. McEvitt is convinced that its value counterbalances this disadvantage. Vomiting has very seldom been troublesome in the cases thus treated, and hence diet problems have been easily solved.

### Death from Accidental Generalised Vaccinia.

Two children were taken to the doctor to be vaccinated. The operation was performed on the elder, aged 3½. It was, however, deemed advisable to postpone the vaccination of the younger child, aged two, until a later date, as traces of eczema were noticed on his face and upper extremities. Three weeks passed, during which the vaccination of the first child followed its normal evolution. On the twenty-first day, however, the unvaccinated child was suddenly taken ill with fever and delirium, and in a few hours pustules appeared on the face and upper limbs, which quickly became confluent, giving the appearance of a generalised vaccinial eruption. After a time, during which severe symptoms were met with, the eruption gradually dried up, only to be followed by the appearance of signs of cerebral infection localised to the psycho-motor and sensory centres of the right hemisphere. Clonic and tonic convulsions of the whole of the left side of the body and face were noticed. Death occurred on the sixteenth day from the onset of the eruption. At the post-mortem examination there were found hæmorrhages of the piamater and cerebral substance, with numerous thrombi in the sinuses. The conclusions which must be arrived at from the study of this case are, according to Dr. Geronne, who reports it in the *Berliner Klinische Wochenschrifte*, that newly vaccinated children must, as far as possible, be prevented from coming into contact with persons suffering from eczema, especially when the latter have not been previously vaccinated, and that in those cases where it is impracticable to separate the newly vaccinated from the eczematous, the greatest care should be taken to prevent all possibility of accidental inoculation by seeing that the vaccinated parts are always kept carefully bandaged.

### The After-effects of Perineorrhaphy.

At a recent meeting of the Société d'Obstétrique de Paris, Dr. Bonnaire drew attention to the effects which a too-perfect restoration of a perineal rupture may cause in a subsequent pregnancy. A woman who had undergone an operation for ruptured perineum was admitted to the Lariboisière maternity when again pregnant and at term. The period of expulsion lasted four hours, her perineum measured seven inches, and as it was commencing to become bloodless in the sagittal line, a medio-lateral episiotomy was performed. The fœtus was immediately expelled *en masse*. On exploring the vagina the posterior wall was found to be transversely ruptured and retracted fairly high up, leaving a ridge marking the edge of the rupture. In addition, a large thrombus was found in the ischio-rectal fossa. The levator ani was ruptured across in the neighbourhood of its bony insertion, and not at its middle, near the vulvo-vaginal orifice, the former reparative operation having rendered this portion of the muscle proof against tearing. The levator ani on one side consequently hung like a curtain in the vagina. The injuries were probably produced owing to the necessity at the time of the previous operation of joining together not only the edges but the surfaces of the ruptured portions of the levator muscle. As a consequence of this joining the muscle becomes a true digastric. If when it descends upon the pelvic floor the fœtal head is unable to pass over this junction of the muscle, the force exerted by the uterine contractions will tend to force it behind the obstruction, *i.e.* into that portion of the floor which lies between the fourchette of the vulva and the coccyx. As the result of this much laceration of the parts will inevitably occur.

### The Nephro-toxic Action of Flesh-meat.

Linossier has recently reported to the Académie de Médecine de Paris some interesting experiments with regard to the nephro-toxic action of various meats. By subcutaneous injection of an aqueous extract of hashed meat he has been able to produce albuminuria in rabbits and guinea-pigs. The minimum dose necessary to cause this condition is very variable even when the same kind of meat is used to prepare the extract, a fact which must be attributed as much to a difference in the renal resistance of various animals as to variations in the toxicity of the meat. Albuminuria appears very quickly after the injection, and only lasts a few hours. It is impossible to cause a typical epithelial nephritis or a permanent albuminuria, even with repeated injections, the animal always dying with marked symptoms of anaphylaxis before such a condition is reached. After contact with natural or artificial gastric juice for two hours the nephro-toxic action of the meat extracts is destroyed, but contact with alkaline solutions does not produce this effect. It would therefore seem that the action of the fluid extract is not due to the extractives contained in the meat, since these are unaffected by gastric juice, but to an inherent property of the albuminous material itself. It is probable that man acquires toleration to the toxic action of meat, but this does not mean that heavy meals can be habitually indulged in with impunity. The accidental and excessive use of meat by a vegetarian would probably be productive of harm, but it is fair to suppose that regular and properly graduated meat diet would be beneficial to a nephritic.

